# Relationship between anesthesia and postoperative endophthalmitis: A retrospective study

**DOI:** 10.1097/MD.0000000000006455

**Published:** 2017-03-24

**Authors:** Hou-Chuan Lai, Wei-Cheng Tseng, Shu-I Pao, Chih-Shung Wong, Ren-Chih Huang, Wei-Hung Chan, Zhi-Fu Wu

**Affiliations:** a Department of Anesthesiology, Tri-Service General Hospital and National Defense Medical Center, Taipei, Taiwan, Republic of China; b Department of Ophthalmology, Tri-Service General Hospital and National Defense Medical Center, Taipei, Taiwan, Republic of China; c Department of Anesthesiology, Cathay General Hospital, Taipei, Taiwan, Republic of China.

**Keywords:** endophthalmitis, inhalation anesthesia, ocular surgery, propofol, topical anesthesia

## Abstract

Previous study showed that patients under general anesthesia (GA) had nasopharyngeal secretions on the face at the end of ocular surgery, especially in propofol-based total intravenous anesthesia (TIVA), it might induce postoperative endophthalmitis. Therefore, we conducted a retrospective study to compare the incidence of endophthalmitis after ocular surgery under topical, inhalation anesthesia, and propofol-based TIVA in our medical center from 2011 to 2015.

A total of 21,032 patients were included, and we evaluated epidemiologic factors, systemic diseases, other ocular pathologic characteristics, complications during the surgery, technique of ocular surgery, method of antibiotic prophylaxis, vitreous culture, and vision outcome in these patients.

Fifteen endophthalmitis cases among 21,032 operations reported, equaling an incidence of 0.071%. The incidence rates under topical, inhalation anesthesia, and propofol-based TIVA were 0.083%, 0.039%, and 0%, respectively (*P* = 0.39). Moreover, the risk of endophthalmitis under GA (0.024%) was significantly lower than topical anesthesia (0.083%) (*P* < 0.001). We also found that elder was the risk factor for endophthalmitis following ocular surgery.

In conclusion, propofol-based TIVA or inhalation anesthesia did not increase the risk of endophthalmitis after ocular surgery. Thus, GA was not a risk factor for postoperative endophthalmitis. By contrast, elder was the risk factor for postoperative endophthalmitis.

## 1 Introduction

Postoperative endophthalmitis is a rare but disastrous complication of ocular surgery, with reported incidence rates of 0.04% to 0.41% in cataract surgery, 0.19% in trabeculectomy, and 0.05% in pars plana vitrectomy.^[1–4]^

Naggle and Cooper^[5]^ reported that about 10% patients under general anesthesia (GA) had nasopharyngeal secretions on the face at the end of ocular surgery, and secretions toward the eye in some patients. Previous studies had showed that propofol anesthesia would increase salivation.^[6–8]^ In our hospital, we also found that some patients had nasopharyngeal secretions toward the eye resulting in contamination of surgical field in propofol-based total intravenous anesthesia (TIVA) in ophthalmic surgery.^[9]^ Besides, Bautista and Keech^[10]^ reported 2 cases under propofol anesthesia with excess nasopharyngeal secretions resulting in surgical contamination in strabismus surgery.

The excess secretions during ocular surgery might induce postoperative endophthalmitis, for this reason, TIVA is not suggested for ocular surgery. However, the incidence of postoperative nausea and vomiting and the need for antiemetics were significantly less in the TIVA patients than in the inhalation anesthesia patients in ophthalmic surgery.^[11]^ Because ostoperative nausea and vomiting will increase intraocular pressure resulting in wound dehiscence and glaucoma.^[12,13]^

Topical anesthesia has become more popular over the past few years and most cataract extractions are performed using this type of anesthesia; however, there may be an association between topical anesthesia and endophthalmitis after cataract extraction.^[14]^

As our best knowledge, a rigorous comparison of the incidence of endophthalmitis after ocular surgery under topical, inhalation anesthesia, and propofol-based TIVA has not yet been performed. Therefore, in this study, we retrospectively investigated the incidence of endophthalmitis after ocular surgery under topical, inhalation anesthesia, and propofol-based TIVA.

## 2 Methods

This retrospective study was approved by the Ethics Committee (TSGHIRB No: 1-105-05-062) of Tri-Service General Hospital, Taipei, Taiwan (Chairperson, Professor Mu-Hsien Yu) on April 28th, 2016. Institutional review board allows waiving the requirement for obtaining informed consent and patient records were anonymized and deidentified prior to analysis. The information was retrieved from the medical records and the electronic database of Tri-Service General Hospital (TSGH; Taipei, Taiwan, Republic of China). We retrospectively analyzed 21,032 patients (American Society of Anesthesiology class I–III) who received elective ocular surgery from January 2011 to December 2015. Records were excluded if data indicated the eye had undergone previous intraocular surgery and age <20 years.

All the patients with a diagnosis code for endophthalmitis using the International Classification of Diseases, Ninth Revision, Clinical Modification codes or similar codes in older records were considered as endophthalmitis: 360.00, purulent endophthalmitis, unspecified; 360.01, acute endophthalmitis; 360.02, panophthalmitis; 360.03, chronic endophthalmitis; and 360.04, vitreous abscess.^[15]^

We retrospectively evaluated the following variables between the cases and the entire study population: demographic factors, regimens of anesthesia, systemic diseases, prophylactic antibiotic regimen, bacterial species of infection, management of the endophthalmitis, surgical procedure, intraoperative complications, and final visual acuity. All methods were performed in accordance with the relevant guidelines and regulations by our institutional review board.

When endophthalmitis was suspected, a vitreous biopsy was performed immediately and sent to the microbiological laboratory for smear and culture and antibiogram analyses. Endophthalmitis was managed according to the recommendations of the Endophthalmitis Vitrectomy Study.^[16]^

In the topical anesthesia, 4 drops topical proparacaine 0.5% combined with 0.2 mL intracameral nonpreserved lidocaine 0.5% were administered to all patients in cataract surgery. Both topical anesthesia and GA patients received 5% povidone–iodine for 5 minutes before surgery.

In the TIVA, anesthesia was induced by using intravenous (iv) fentanyl (2 μg/kg) and 2% lidocaine (1.5 mg/kg). Continuous infusion of propofol (Fresfol 1%) was delivered subsequently by using Schneider kinetic model of target controlled infusion (Fresenius Orchestra Primea; Fresenius Kabi AG, Bad Homburg, Germany) with the effect-site concentration of 4.0 μg/mL rocuronium (0.6 mg/kg) was administered when patients lose of consciousness, followed by tracheal intubation. Anesthesia was maintained by using target controlled infusion with propofol effect-site concentration 3 to 4 μg/mL and a 50% oxygen with flow of 1 L/min.^[11,17–22]^

In the inhalation anesthesia, the patients were induced with fentanyl (2 μg/kg, iv), 2% lidocaine (1.5 mg/kg, iv), and propofol (1.5–2 mg/kg, iv). When patients lose of consciousness, 0.6 mg/kg of rocuronium iv was administered, followed by endotracheal intubation. Anesthesia was maintained by using 8% to 12% desflurane (inhaled concentration) in a 100% oxygen with flow of 0.3 L/min.^[11,17–22]^

Maintenance of the propofol or desflurane concentration was adjusted to keep mean blood pressure at 80 to 100 mm Hg or within 20% of baseline. The end-tidal carbon dioxide pressure was maintained at 35 to 45 mm Hg by adjusting the ventilation rate and maximum airway pressure below 30 cmH_2_O. Repetitive bolus injections of rocuronium 5 to 10 mg iv were prescribed as required throughout the procedure.^[11,17–22]^

At the end of surgery, propofol or desflurane was discontinued, and the lungs were ventilated with 100% oxygen at a fresh gas flow of 6 L/min. Reversal of neuromuscular function was achieved by administrating neostigmine (0.03–0.04 mg/kg, iv) with glycopyrrolate (0.006–0.008 mg/kg, iv) once spontaneous breathing returned to prevent residual paralysis. When the patient regained consciousness by name with spontaneous and smooth respiration, the endotracheal tube was removed and the patient was sent to the postanesthesia care unit for further care.^[11,17–22]^

Data are presented as the mean and standard deviation, number of patients, or percentage. Demographic and surgical time was compared using Student *t* tests or Mann–Whitney *U* test while the data were not normally distributed. Categorical variables were compared using chi-square test. Statistical significance was accepted for 2-tailed *P* values of <0.05. The statistics was performed by using SigmaStat 3.5 for Windows.

## 3 Results

The summary of patient's characteristics and surgical procedure is shown in Table 1. The age of the topical anesthesia group (60.1 ± 16.2 years) was significantly older than GA group (55.2 ± 16.3 years) (*P* < 0.001). Besides, the surgical time of topical anesthesia group (33.3 ± 8.1 min) was shorter than GA group (129.2 ± 39.9 minutes) (*P* < 0.001).

**Table 1:**
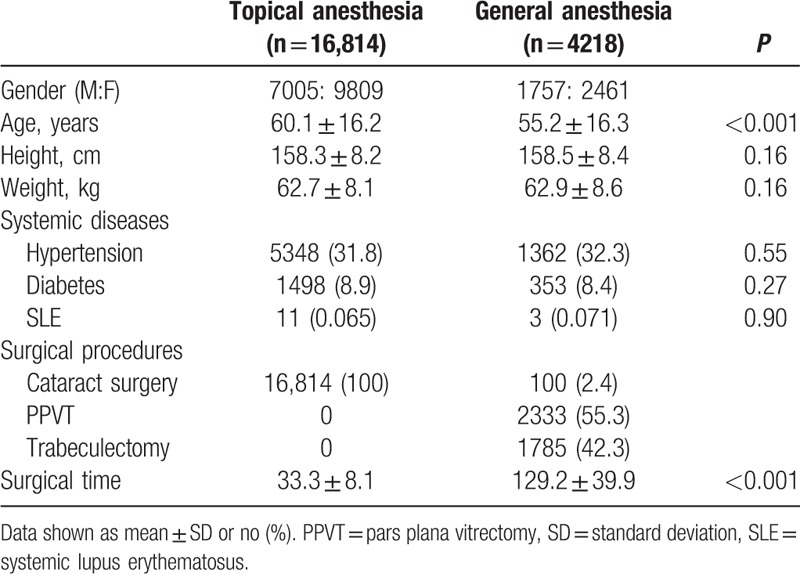
Patient's characteristics and surgical procedures.

Analysis of the medical records revealed 15 postoperative endophthalmitis cases among 21,032 operations, indicating a postoperative endophthalmitis incidence of 0.071%. The age of the entire ocular surgery was 59.1 ± 16.2 years. Postoperative endophthalmitis developed in significantly older patients with a 67.9 ± 12.5 years (*P*�=�0.04), besides, a significantly higher rate of endophthalmitis with systemic lupus erythematosus (SLE, 6.7%) than entire ocular surgery (0.067%) (*P* < 0.001). Table 2 shows the summary of clinical findings and specifications of patients with endophthalmitis.

**Table 2:**
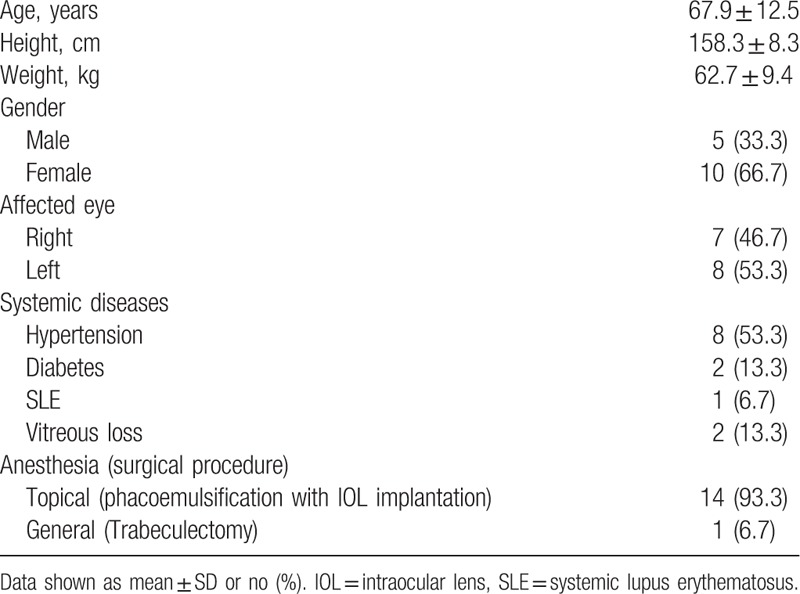
Summary of demographic data of all endophthalmitis cases.

Endophthalmitis occurred in 14 eyes (93.3%) after phacoemulsification with intraocular lens implantation under topical anesthesia, and 1 eye (6.7%) after trabeulectomy under inhalation anesthesia. Table 3 shows the rate of endophthalmitis according to different anesthesia. The incidence of endophthalmitis (14/16,814, 0.083%) was present in the cataract surgery under topical anesthesia, whereas 1 case suffering from postoperative endophthalmitis was noted in the 2571 operations under inhalation anesthesia (0.039%). No endophthalmitis was found in the 1647 patients under propofol-based TIVA. The incidence rates of postoperative endophthalmitis showed no significantly differences among the 3 anesthesia (*P* = 0.39). However, the risk of endophthalmitis under GA (0.024%) was significantly lower than topical anesthesia (0.083%) (*P* < 0.001).

**Table 3:**
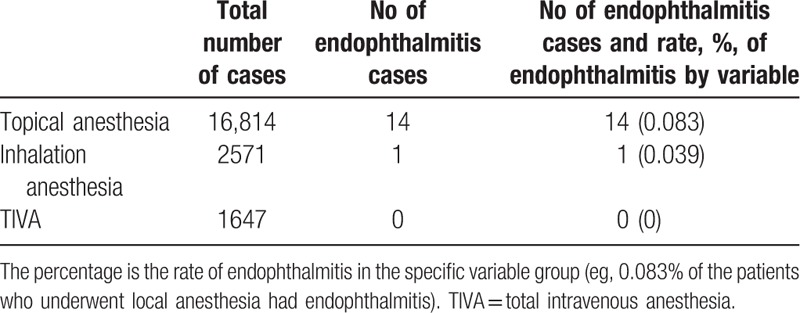
Rate of endophthalmitis according to different anesthesia.

All patients suffering from postoperative endophthalmitis received perioperative antibiotics. Endophthalmitis occurred in 14 eyes (93.3%) receiving intracameral cefuroxime in cataract surgery, whereas 1 eye (6.7%) by using subconjunctival gentamycin in trabeculectomy (Table 4).

**Table 4:**
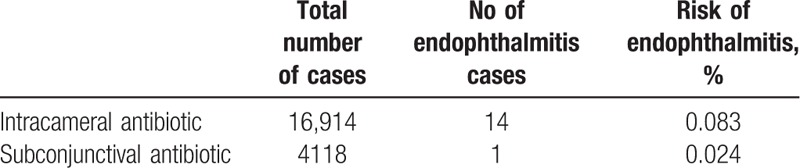
Analysis for prophylactic antibiotic use.

Among all vitreous samples obtained from endophthalmitis cases, 9 samples (60%) showed positive culture results; remaining 6 cases (40%) with a clinical diagnosis of endophthalmitis showed negative culture results (Table 5). The final visual acuity was better in patients with negative culture results. In contrast, eyes with coagulase-negative staphylococci, *Mycobacterium chelonae*, or *Trichophyton* spp had poor final vision (Table 5).

**Table 5:**
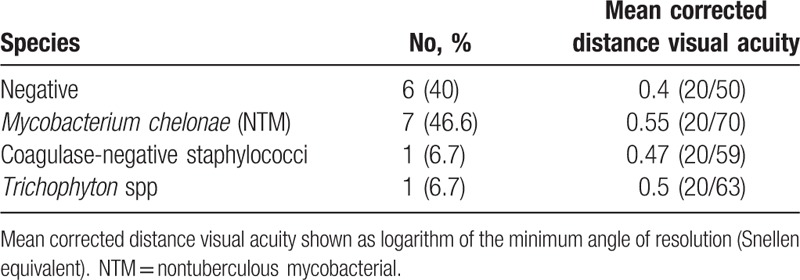
Culture results and final visual outcomes.

## 4 Discussion

This study is one of the largest studies of endophthalmitis occurring after ocular surgery under different anesthesia. The major findings in this retrospective study showed that propofol-based TIVA or inhalation anesthesia did not increase the risk of endophthalmitis after ocular surgery.

The patient's ocular flora or microorganisms that have colonized surface eye structures (eyelids and conjunctiva) are the usual causes of infection.^[4]^ The most common cultured microorganisms are gram-positive coagulase-negative cocci (about 70% of cases), with *Staphylococcus epidermidis* the most prevalent and streptococci species seen less frequently.^[4]^ Thus, isolating the eyelids and eyelashes from the surgical field is crucial. Durand^[23]^ reported that coagulase-negative staphylococci are the most common causes of postcataract endophthalmitis, and these bacteria and viridans streptococci cause most cases of postintravitreal antivascular endothelial growth factor injection endophthalmitis. In the current study, however, *M chelonae* was the most prevalent isolated bacteria (7/15 patients, 46.6%). It might be resulting from the presence of an implant in or around the globe (prosthetics placed after enucleation, nasolacrimal silicone stents, scleral buckles, contact lenses, glaucoma implants, and punctal plugs) or immunocompromised states such as SLE and diabetes.^[24]^ The most accepted practice to prevent endophthalmitis is the topical use of 5% povidone–iodine in the conjunctival sac before surgery as our practice.^[4]^

Our finding was consistent with previous study reporting that GA did not increase the incidence of endophthalmitis after ocular surgery.^[5]^ Although, the TIVA patients may produce more nasopharyngeal secretions than inhalation anesthesia and cause postoperative endophthalmitis; however, we did not find the correlation. Therefore, we concluded that the greater nasopharyngeal secretions in GA were not the major risk factor in postoperative endophthalmitis following ocular surgery. It might be due to 2 reasons. First, according to surgical procedures, we administered postoperative injection of subconjunctival antibiotics to 97.6% GA patients in our hospital. It might prevent postoperative endophthalmitis.^[4]^ Second, GA patients keeping their eyes still during surgery, hence the corneal incisions might not reach the conjunctival fornix, eyelids, and eyelashes, which can harbor microorganisms.^[14]^

GA can be administered using inhalational anesthetics, intravenous medications, or most frequently a combination of both. All of these forms of anesthesia have been found to result in immunosuppression and postoperative infections.^[25,26]^ In contrast, regional anesthesia causes less immunosuppression than GA.^[27]^ In this study, however, GA did not increase the risk of endophthalmitis after ocular surgery. It might be due to less surgical stress and shorter surgical time in ocular surgery than other major operations. In addition, Garcia-Arumi et al^[14]^ reported that topical anesthesia is a possible risk factor for the development of endophthalmitis. In our study, the incidence of endophthalmitis under topical anesthesia was 0.083%, higher than 0.024% under GA, consistent with the above study.^[14]^ The mechanism might be that topical anesthesia patients cannot keep their eyes still, therefore, the corneal incisions might reach the conjunctival fornix, eyelids, and eyelashes, which can harbor microorganisms.^[14]^

Older age was a risk factor for endophthalmitis,^[28,29]^ in our study, endophthalmitis patients were 67.9 ± 12.5 years, consistent with 66.9 ± 15.3 years of previous Asian study.^[29]^ Two possible causes would explain this finding. First, with aging, a cataract hardens and zonules weaken, so the operation will be more prone to complication, and endophthalmitis is more prevalent in complicated cataract surgery. Second, more bacteria are present in the conjunctiva of older patients compared with that of younger patients.^[28]^

This study also demonstrated a statistically higher rate of endophthalmitis in SLE patients. One (6.7%) of our patients with endophthalmitis had SLE, and it might induce infectious endophthalmitis.^[30]^ However, we only revealed 1 patient, therefore, a large study was needed.

Nam et al^[29]^ reported that the most common systemic disease in endophthalmitis was hypertension (40.4%). A total of 53.3% of our endophthalmitis patients having hypertension was higher than the total ocular surgery (31.9%); however, no significant difference was found in our study (*P* = 0.075).

Recent study also had mentioned diabetes as an independent risk factor of endophthalmitis.^[28]^ There was 13.3% of our endophthalmitis patients had diabetes mellitus, it was consistent with 14.3% of recent study^[28]^; however, we did not find the statistically significant difference with the total ocular surgery (8.8%) (*P* = 0.54).

Antibiotic prophylaxis overall showed a reduction in risk.^[2,4]^ Short-term pretreatment of topical antibiotics such as neosporin, levofloxacin, or moxifloxacin reduced the conjunctival microbial burden in previous reports.^[31–34]^ Besides, postoperative injection of subconjunctival antibiotics might prevent endophthalmitis.^[2,4]^ However, there was no consensus in the literature for the effect of prophylactic antibiotics to prevent endophthalmitis.^[28]^ Therefore, we suggested that further investigation is required to evaluate completely the effectiveness of different kinds and routes of prophylactic antibiotics on cases of postsurgical endophthalmitis. Isolating the organism was the mainstay of the antibacterial therapy of endophthalmitis. Only 9 of 15 vitreous culture results were positive. Previous studies reported gram-positive bacteria as the most common isolated bacteria.^[35]^ In this study, a high cross-species transmission positive rate for *Mycobacterium* following cataract surgery was consistent with the recent report.^[24]^

The role of vitreous loss in postoperative endophthalmitis was first identified by Javitt et al^[36]^ in 1991 and has been confirmed in the recent subsequent report.^[28]^ Vitreous loss was identified in association with 13.3% of endophthalmitis cases in this study, comparable with 18% of the recently published study.^[28]^

There were limitations in this study. First, it was hard to distinguish whether experienced surgeons or inexperienced residents made the surgeries from medical records. Previous studies^[15,37,38]^ reported that longer surgical experience and a higher annual volume of surgery decreased the risk of postoperative endophthalmitis. Second, we could not distinguish between intraocular hydrophobic lenses and hydrophilic lenses. Baillif et al^[39]^ found greater bacterial adherence to hydrophobic lenses compared with hydrophilic lenses. Finally, the different characteristics such as age, surgical time, and wound sites were different in the topical and GA groups, because they had different diseases and received different operations, and these might be confounding factors.

In conclusion, the propofol-based TIVA or inhalation anesthesia did not increase the risk of postoperative endophthalmitis after ocular surgery even the excess nasopharyngeal secretions during surgery. Thus, GA was not a risk factor for postoperative endophthalmitis. By contrast, elder was the risk factor for postoperative endophthalmitis.
